# Translating radiological research into practice—from discovery to clinical impact

**DOI:** 10.1186/s13244-023-01596-2

**Published:** 2024-01-17

**Authors:** Marion Smits, Andrea Rockall, Stefan N. Constantinescu, Francesco Sardanelli, Luis Martí-Bonmatí

**Affiliations:** 1 Department of Radiology & Nuclear Medicine, Erasmus MC – University Medical Centre Rotterdam, Rotterdam, The Netherlands; 2 Medical Delta, Delft, The Netherlands; 3 Department of Surgery and Cancer, Faculty of Medicine, Imperial College London, London, UK; 4 Department of Radiology, Imperial College Healthcare NHS Trust, London, UK; 5 Ludwig Institute for Cancer Research, Brussels, Belgium; 6 de Duve Institute, Université Catholique de Louvain, Brussels, Belgium; 7 WEL Research Institute, WELBIO Department, Wavre, Belgium; 8 Ludwig Institute for Cancer Research, Nuffield Department of Medicine, Oxford University, Oxford, UK; 9 Department of Biomedical Sciences for Health, Università Degli Studi Di Milano, Milan, Italy; 10 Unit of Radiology, IRCCS Policlinico San Donato, San Donato Milanese, Milan, Italy; 11 Department of Radiology and GIBI230 Research Group On Biomedical Imaging, Hospital Universitario y Politécnico La Fe and Instituto de Investigación Sanitaria La Fe, Valencia, Spain

**Keywords:** Evidence-based medicine, Radiology, Research, Methods, Health technology assessment

## Abstract

At the European Society of Radiology (ESR), we strive to provide evidence for radiological practices that improve patient outcomes and have a societal impact. Successful translation of radiological research into clinical practice requires multiple factors including tailored methodology, a multidisciplinary approach aiming *beyond* technical validation, and a focus on unmet clinical needs. Low levels of evidence are a threat to radiology, resulting in low visibility and credibility. Here, we provide the background and rationale for the thematic series *Translating radiological research into practice—from discovery to clinical impact*, inviting authors to describe their processes of achieving clinically impactful radiological research. We describe the challenges unique to radiological research. Additionally, a survey was sent to non-radiological clinical societies. The majority of respondents (6/11) were in the field of gastrointestinal/abdominal medicine. The implementation of CT/MRI techniques for disease characterisation, detection and staging of cancer, and treatment planning and radiological interventions were mentioned as the most important radiological developments in the past years. The perception was that patients are substantially unaware of the impact of these developments. Unmet clinical needs were mostly early diagnosis and staging of cancer, microstructural/functional assessment of tissues and organs, and implant assessment. All but one respondent considered radiology important for research in their discipline, but five indicated that radiology is currently not involved in their research. Radiology research holds the potential for being transformative to medical practice. It is our responsibility to take the lead in studies including radiology and strive towards the highest levels of evidence.

**Critical relevance statement** For radiological research to make a clinical and societal impact, radiologists should take the lead in radiological studies, go beyond the assessment of technical feasibility and diagnostic accuracy, and—in a multidisciplinary approach—address clinical unmet needs.

**Key points**

• Multiple factors are essential for radiological research to make a clinical and societal impact.

• Radiological research needs to go beyond diagnostic accuracy and address unmet clinical needs.

• Radiologists should take the lead in radiological studies with a multidisciplinary approach.

**Graphical Abstract:**

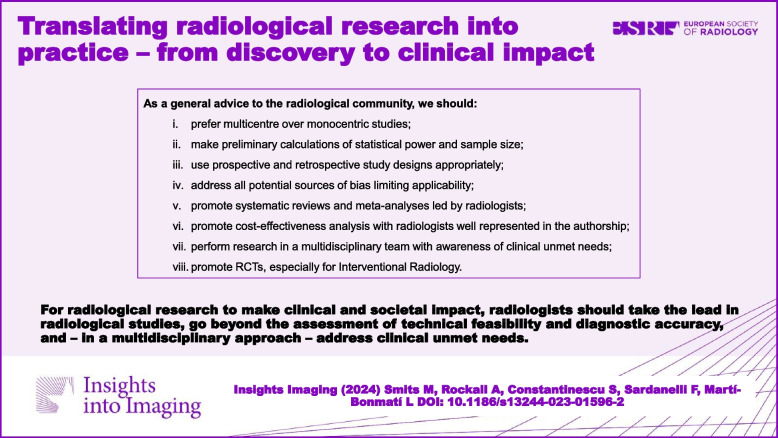

## Introduction and rationale

Clinical research seeks to provide scientific evidence for diagnostic and therapeutic practices with the aim of improving patient outcomes and having a beneficial societal impact [1]. In their 2010 article on evidence-based medicine (EBM) for radiology, Sardanelli et al. reported the previously introduced hierarchy of studies on diagnostic tests, ranging from level 1 (technical performance) to level 6 (societal impact) [2, 3]. The authors were hopeful that radiological research, which was relatively slow in its uptake of EBM, would catch up. At the time, it was estimated that less than 10% of standard radiological procedures were supported by high-level evidence [4]. Indeed, over a decade later, many impactful radiological studies have now been published that truly changed clinical practice, including the management of suspected prostate cancer [5], treatment of stroke [6], and screening for breast cancer [7, 8] to name a few. What many of these highly impactful studies have in common is that they are the result of a fruitful collaboration of the various disciplines involved in the diagnosis and treatment of these particular diseases.

At the same time, a high rate of radiological research studies continues to focus on technical or diagnostic performance only, often using a retrospective study design including selected cohorts, without following on with the prospective validation [9, 10]. The assessment of technical and diagnostic performance should not be trivialised, as these are crucial first steps in assessing radiological performance and our focus on these is inherent to the technical nature of our profession [2]. Importantly, the safety of radiological procedures also needs to be assessed, as an initial step with technical performance (e.g. for CE marking) and thereafter as part of post-market surveillance. But the story does not end with technical and diagnostic performance. A clear distinction should be made between diagnostic benefit and benefit to the (individual) patient and eventually also to society, whether this is a change in management, outcome, and/or costs [3]. It is not a given that better technical or diagnostic performance actually changes patient management or outcome or has any impact on the societal level [11]. Also, prospective validation of the results of these studies is mandatory to fully evaluate the impact of the research on real-world practice, acknowledging that such work is highly challenging due to the costs and time required. As these domains are typically outside the general scope of diagnostic radiology, they receive (too) little attention in the radiological community.

The frequent lack of assessment of therapeutic and outcome impact by radiological studies not only hampers the penetration of new radiological developments into clinical practice and particularly into guidelines, but also impedes our strength, visibility, and impact as radiologists in the daily care of patients. An important indicator of this is the finding that out of 867 systematic reviews on diagnostic and interventional imaging between 2001 and 2010, only 330 (38%) were published by an imaging specialist as the first or last author. In other words, a large majority of systematic reviews on our specialty were led by non-imaging specialists [12]. What is more, the quality of those reviews not including imaging specialists among the authors was significantly lower than that of those including them [12]. This means that lower-quality systematic review publications dominated, which is detrimental to our credibility and impedes the introduction of advanced imaging techniques into clinical routine. In the recent NICE guideline on brain tumour management, for instance, all studies on perfusion MRI were considered low or very low-quality evidence [13]. Recommendations could therefore only be based on the committee’s clinical experience rather than scientific evidence, reducing their potential clinical impact.

There are multiple initiatives to assess and improve the quality of radiological research. Reporting guidelines such as STARD [14] or TRIPOD [15] specifically address diagnostic studies. But for radiological research to make a clinical impact, we also need to go *beyond* the assessment of technical and diagnostic performance, and address clinical unmet needs, connecting with referring/treating physicians to ensure their support and uptake into a clinical setting. In other words, our focus should not only be on the image, but on the full clinical setting, patient outcome, and eventually on society as a whole. The European Institute for Biomedical Imaging Research (EIBIR) established the European Network for the Assessment of Imaging in Medicine (EuroAIM) to systematically assess radiological technology and seek evidence for its best use in clinical practice. A number of EuroAIM initiatives were dedicated to critical appraisal of the quality of imaging guidelines using the AGREE II tool (see for example [16, 17]). Its application provides insight into the unmet radiological needs in clinical guidelines, for example, showing limitations in the “applicability” domain. It is imperative that we, as radiologists, truly take the lead in addressing these unmet needs by initiating and performing high-quality studies and ensuring their clinical implementation. More broadly, studies led by radiologists as principal investigators should approach difficult questions with original hypotheses and novel technologies, starting from fundamental questions in the field. This is not always self-evident from the commonly supportive role we have in a clinical setting, but even for aspects such as patient inclusion, we as radiologists can and should consider ourselves responsible to truly take the lead in such studies.

## Objective

The objective of this article is to provide the background and rationale for the thematic series on *Translating radiological research into practice—from discovery to clinical impact*, which invites authors to describe the process of achieving clinically impactful radiological research as guidance and inspiration to the radiological research community. We will define impact and describe the specific challenges for relevant radiological research and those aspects that make diagnostic research unique in comparison to other clinical research. We will, furthermore, explain the various methodological aspects that result in impactful radiological research, specifically aiming to go *beyond* diagnostic accuracy. We will also highlight the importance of multidisciplinary research and provide examples of currently unmet clinical needs which provide potential areas of research where high impact could be achieved.

## What is impact?

Each intervention undertaken in a patient’s care pathway will have an impact, which may be positive or negative, intended or unintended [18]. The impact of a radiological ‘intervention’ may be straightforward, such as influencing the start of treatment, for example, following the diagnosis of a pulmonary embolism, detection of osteoporosis, or screening for cancer. Impact evaluations may include a wide range of events, such as diagnosis, definition of or change in treatment plan, and selection of patients for trials. However, the impact of an imaging investigation may be difficult to tease out when considering complex scenarios affecting patient outcomes, due to multifactorial interacting events such as the patient’s performance status and differing treatment regimes.

Certainly, the exponential increase in requests for imaging investigations to guide treatment planning decisions is a form of evidence of the positive impact of imaging on patient pathways. Imaging investigations that are widely implemented into clinical practice are clearly valued by referring physicians for treatment planning. However, in order to measure impact more directly, it is necessary to formulate a clear question that will allow effective impact evaluation. For example, ‘what is the impact of MRI on patient selection for prostate biopsy?’. In this example, several kinds of impact may be considered, such as the following:

- Did MRI increase the proportion of positive biopsies of significant tumours compared to ultrasound (US) alone?
- Did MRI reduce the proportion of biopsies of non-significant tumours or abnormalities compared to US alone?

These questions allow measurable impact evaluation of the intervention. A clear positive impact of the imaging investigation has resulted in widespread change in clinical practice and adoption of the investigation into clinical guidelines. In addition, impact can be measured by the cost savings based on the reduction of the number of negative biopsies.

Clinical impact of radiological imaging may also be evaluated by patient preference, for example, in the case of MRI guiding fertility-preserving surgery in gynaecological cancer. In this case, the outcomes can be measured for both fertility events and cancer events. Patient preferences and the impact of imaging investigations on quality of life need to be addressed. Imaging findings can furthermore impact patient selection for clinical trials, where image-based eligibility criteria are used (e.g. presence of tumour above a certain size) and for stratification of patients into different study arms (for example, patients with or without metastatic disease on imaging). Large-scale imaging studies in non-patient populations can have an impact through providing insight into disease aetiology and risk factors such as stroke [19] or dementia [20].

Imaging to screen for relevant diseases may have a much wider societal impact, with a clear benefit to patient survival rates as in breast cancer-screened populations [21]. However, it is important to conduct appropriate large-scale screening trials; as the cost of population screening is high, there may be negative impacts on patient quality of life, and the impact on patient survival may not be present, as demonstrated in the UKCTOCs study in ovarian cancer screening [22].

Techniques to measure the value of imaging investigations, including health technology assessment, are even more important at this time of implementation of artificial intelligence (AI) in radiology [23]. The value of imaging in the patient experience is also an essential aspect of measuring our impact on value-based care [24, 25].

## Importance of a multidisciplinary approach

Science and medicine are complex. No simple solution exists to understand the fundamentals of the biological processes and the use of current and future targeted treatments. To improve results, researchers also have to properly define the clinical pathway and outcomes to address, connect relevant technical and clinical aspects, access data repositories, understand other members’ skills, guarantee enough time to the research project, follow accepted checklists and guidelines, search for evidence-based knowledge, and develop common research skills. A multidisciplinary approach is mandatory not only between different specialties within medicine, but also with other disciplines such as data scientists, physicists, or computational engineers. Particularly in this era of precision medicine, where big data and AI are key to building robust predictive models integrating imaging and non-imaging data, a multidisciplinary approach is key also at the level of data collection. While there are multiple efforts to store and publicise such diverse datasets in the form of biobanks, only a minority have dedicated functionality for imaging data [26]. As an important effort to help imaging researchers to share or use imaging data, the ESR and EIBIR have set up an imaging biobank catalogue with descriptions of imaging biobanks and image collections in order to advertise them towards the research community (https://molgenis.eibir-edc.org/menu/main/app-molgenis-app-biobank-explorer#/).

Involving treating physicians is essential for the implementation and uptake of radiological advances in new guidelines. All relevant papers do have to finally address clinically relevant questions and unmet needs to improve how patients are diagnosed and treated. Even if the focus of a research work is technical, the foreseen clinical improvements must be highlighted, which can only be achieved by involving all relevant disciplines, each with their specific expertise. In a comparative study of various handbooks of guideline development, a multidisciplinary panel was considered a key aspect for issuing guidelines [27], to ensure input from all relevant stakeholders and timely identification of concerns with implementation [28]. It should be noted that developing guidelines (following the proper GRADE II methodology) requires a substantial time (1 year, minimum) and financial investment. Online meeting tools, however, have greatly facilitated cross-institutional/national and multidisciplinary collaboration and the ESR’s and subspecialty societies’ networks could provide an ideal starting point for such initiatives.

## Unmet clinical needs

To assess the impact of radiological developments and the currently unmet imaging needs, a survey was sent to 23 non-radiological clinical societies and associations with whom the ESR has a memorandum of understanding or had any other formal contact in past years. The survey consisted of 5 open-answer questions (Table 1). Twelve responses were received from 11 societies (1 society provided 2 responses). The majority of respondents (6/11) were in the field of gastrointestinal/abdominal medicine; others were in the field of musculoskeletal (3/11), oncological (1/11), and diabetes (1/11) care. The implementation of CT or MRI techniques for disease characterisation, detection and staging of cancer, and surgical planning, together with radiological interventions were mentioned as the most important radiological developments in the past years (Table 2). The perception was that patients were not always aware of the impact of these developments, and when they were, the awareness was mostly modest (Table 2). Unmet clinical needs were mostly in the context of staging for various forms of (gastrointestinal) cancer, early and differential diagnosis, microstructural and/or functional assessment of tissues and organs, and the visualisation of implants (Table 3). All respondents but 1 considered radiology (very) important for clinical research and their discipline, but 5 indicated radiology was currently not involved in their clinical research (3 in gastrointestinal/abdominal medicine, 1 in diabetes care, and 1 in musculoskeletal disease).

**Table 1:** Survey questions

1. Which society and clinical discipline do you represent?
2. What do you consider the *most important radiological development* in the past years within your area of practice?
a. How did this impact your area of practice?
b. Was this impact perceived by patients and if so, how much?
3. Which are the main clinical issues in your area of practice where radiological imaging falls short and why (maximum 3)?
4. How do you rate the role of radiology for clinical research in your discipline?
5. Are radiologists involved in the study design of clinical research that includes radiological examination?
a. If yes: how? (for instance, is there a specific working group/committee dedicated to imaging, are radiologists actively involved in your society?)

**Table 2:** Most important radiological developments^a^

Development	Impact	Patient perception
Endoscopic ultrasound	Extra-gastrointestinal biopsies and stenting	Yes (a lot)
Tools to provide prognostic biomarkers in pancreatic cancer	Still experimental	Not a lot
Radiology-guided interventions	Improved postoperative management	Yes (little)
Study of the intestine by MRI/CT with contrast agents or small intestine contrast ultrasonography (SICUS), mainly for inflammatory bowel disease management	For diagnosis, evaluation of extension and activity of inflammatory bowel disease and for evaluating response to treatments in Crohn’s disease	Yes
MRI for rectal, proctology, and abdominal pathology	Relying on MRI for rectal cancer (stage, follow, etc.), proctology (fistula, sepsis, tumour, etc.), and abdominal pathology (such as inflammatory bowel disease)	Uncertain, but many people have seen the advances in MRI and asked for it
MRI, contrast-enhanced spectral mammography (CESM)	Improved cancer detection	Yes, even if it involved radiological techniques that required contrast agents and take longer than the conventional ones
Software for pre-operative planning and patient-specific instrumentations for surgery	Invaluable aid for shoulder arthroplasty pre-op study and planning	Probably not
3D weight-bearing CT	Better orthopaedic surgical planning	Probably not
Image-based robotic hip and knee arthroplasty	Image-based robotic surgery has improved the pre-operative dynamic 3D planning, accuracy, and soft tissue balancing of hip and knee arthroplasty, providing better outcomes	The demand for robotic arthroplasty is increasing steadily and with increased patient satisfaction and long-term implant survivorship
MRI arthrography to better detect for instance pathologies of the biceps tendon and labrum acetabuli	Detection of soft tissue injuries and combined pathologies such as anterior cruciate ligament and posterolateral or anterolateral corner. Identification of labrum pathologies in the hip	More specific approach in terms of treatment, either conservative or surgical
Replacement of CT with entero-MRI in the assessment of disease activity and bowel damage	Dramatic change in practice, provided MRI is easily accessible	Not necessarily

^a^Responses from 11 of 23 non-radiological clinical societies and associations

**Table 3:** Main clinical issues where radiological imaging falls short^a^

Gastrointestinal	Musculoskeletal	Oncological/general
Staging of cancer (pancreatic, oesophageal)	Assessment of meniscus healing after meniscus repair	Early diagnosis/early cancer imaging or screening
Proctology	Assessment of healing after rotator cuff repair	Nodal disease
Diagnosis of the nature of the biliary strictures	Cartilage assessment especially in the knee	Cost and accessibility of in particular advanced imaging techniques, limiting the availability of cutting-edge imaging for certain populations
Lack of imaging diagnostic tools in functional disease	Anterior impingement	Assessment of functional changes in tissues or cells
ERCP still provides rudimentary info	Imaging of body parts with a non-MRI compliant orthopaedic implant in place	Microvascular changes
Differential diagnosis of primary liver cancer	MR imaging of body parts with an orthopaedic implant in place	
Chronic or acute pancreatitis imaging	Charcot vs osteomyelitis	
Assessment of evolution of perianal lesions in Crohn’s disease		
Assessment of intestinal fibrosis		
Prediction of intraductal papillary mucinous neoplasm (IPMN) progression		

^a^Responses from 11 of 23 non-radiological clinical societies and associations

## Practical and methodological challenges and recommendations

There are several well-recognised challenges for radiologists trying to conduct imaging research as chief or principal investigators, rather than being in a supportive role, as is commonly the case in clinical trials with imaging used for measurement of treatment effect. Understanding these challenges may help to develop strategies to successfully undertake radiology-led research.

1. Eligible patients may attend outpatient clinics led by referring clinicians who may not suggest taking part in an imaging study, leading to difficulties with recruitment, with patients slipping through. In addition, informed consent can be challenging if the research coordinator or referring clinician does not fully understand the suggested imaging intervention. Radiologists may not have sufficient flexibility to be present in the clinics to recruit and consent patients, especially when only few patients may be eligible per clinic. It becomes essential to employ a research coordinator to attend clinics for patient identification.
2. The treating physician may not be fully supportive of imaging research, thus being an obstacle to present the study to the patient in a positive way to ensure recruitment. There may be competing studies led by treating physicians or indeed they may wish to lead the radiological research themselves, leading to a turf battle.
3. There may not be sufficient flexibility on scanners to readily fit research examinations into a busy clinical schedule. If a research sequence is being added to a standard-of-care scan, there may not be sufficient flexibility in booking slots to allow for ad hoc extension to scan times.
4. Even when setting up a prospective, quality-assured imaging protocol, there can be difficulties in ensuring uniformity of scan acquisition across multiple scanners, multiple sites, or multiple operators. Site training and quality assurance are essential but can be difficult, with so many radiographers involved in scanning patients over several years in multicentre prospective studies [29]. This may lead to difficulties with reproducibility and data variability across a study, negatively impacting the power of the study.
5. Traditional study methodologies are not necessarily well-suited for conducting medical imaging research. A lack of awareness of methodological considerations regarding radiological research carries the risk of inappropriate study design by researchers on the one hand or—unjustified—rejection by reviewers in case a non-traditional yet appropriate method is applied.

Several methodological issues and challenges are specific and sometimes unique to radiological research. One first issue is the only indirect relationship between radiological imaging and treatment, with the obvious exception of interventional radiology. This means that between our diagnoses and patient outcomes, there is a large spectrum of options for confounding interventions and events, for instance, in oncology medical therapy, surgery, radiation therapy (and combinations thereof) and the personal biological status, including genetic and epigenetic factors. Thus, to measure the outcome effect of radiological imaging is the first big methodological challenge.

Well related to this point is the lack of awareness regarding the type of evidence needed for recommending radiological diagnostic procedures in multidisciplinary guidelines. The widely endorsed GRADE system for evaluating the quality of scientific evidence and strength of guideline recommendations [30] requires randomised controlled trials (RCTs) for obtaining the highest levels of evidence, while in fact, RCTs are not always needed [31]. According to EBM, physicians should choose the test showing the best compromise between sensitivity and specificity in the particular clinical scenario, e.g., for patients presenting with symptoms or signs of a disease high sensitivity may be desired to determine a treatable cause (e.g., identifying intracranial vessel occlusion in a patient with neurological symptoms); for staging of an already diagnosed disease, high specificity may be needed to avoid withholding treatment by inadvertent over-staging (e.g. excluding brain metastasis prior to start of systemic treatment) [32]. Sometimes, the entire story is forgotten: RCTs are a surrogate of intraindividual trials that are not possible for comparing different therapies. In fact, when intra-individual trials are possible, e.g. for ophthalmological disease (therapy 1 for the right eye, therapy 2 for the left eye) or dermatological disease (therapy 1 on the right arm, therapy 2 for the left arm), they are the best way, because of minimising the patients’ variability, so also reducing the sample size. Conversely, we—as radiologists—should always ask for RCTs to recommend new screening strategies, to avoid biases inherent to screening (length bias and lead time bias) [33] and to reach the highest level in the hierarchy of studies of diagnostic tests, up to the societal level (e.g. reduction of mortality as an effect of secondary disease prevention) [34].

A challenge is that the higher the levels of evidence we want to obtain, the higher the costs, the longer the time, and the more complex the research organisation we need. Radiology departments commonly do not have their own research nurses, data managers, or statisticians. The work is mostly done by clinical radiologists who also dedicate time to research or by radiology residents. The oft-reported burnout among radiologists due to excessive clinical workload could further impact research activities in radiology departments [35–37]*.* Even when PhD students are involved, we should consider that they frequently pursue a clinical rather than a scientific career after obtaining their PhD title. We need research professionals *inside* radiology departments, the same being true for data scientists and AI experts, to promote cross-fertilisation [38].

As a general advice to the radiological community, we should:

i. Prefer multicentre over monocentric studies
ii. Make preliminary calculations of statistical power and sample size
iii. Use prospective and retrospective study designs appropriately
iv. Address all potential sources of bias limiting applicability
v. Promote systematic reviews and meta-analyses led by radiologists
vi. Promote cost-effectiveness analysis with radiologists well represented in the authorship
vii. Perform research in a multidisciplinary team with awareness of clinical unmet needs
viii. Promote RCTs, especially for interventional radiology

The last recommendation is an absolute need. Radiologists often invented and proposed procedures that then became clinical practice in the hands of other professionals (e.g. ultrasound for obstetrics/gynaecology, neurology, vascular surgery, urology, gastroenterology [39]). In the era of overdiagnosis (also related to an increasingly ageing population), it is important to minimise the impact of invasive procedures for confirming diagnosis or initiating treatment. Interventional radiology, being minimally invasive and already playing an increasing role in oncology, can substantially contribute to this objective [40].

## Conclusion

Successful translation of radiological research into clinical practice requires multiple factors including a sound but at the same time tailored methodology, a multidisciplinary approach aiming *beyond* technical validation, and a focus on unmet clinical needs. Low levels of evidence are a threat to radiology through limited impact and low uptake in guidelines, resulting in low visibility and low credibility of our profession. While radiologists are generally considered important for clinical research, they do not always have a formal role in research involving radiological examinations performed by non-radiological clinical societies. It is our responsibility as radiologists to take the lead in radiological studies and strive towards the highest levels of evidence and reproducibility for the added value of the advances of our discipline. Last but not least, research led by radiologists holds the potential to illuminate areas that go beyond the initial questions and impact medicine more broadly. In this thematic series, we present the factors, not always well-known, behind the success of impactful radiological studies to provide guidance for future radiological research.

## Data Availability

The survey results are in full and available with the first/corresponding author (MS) and the ESR office. For reasons of confidentiality and privacy, these are not made publicly available.

## Abbreviations

AI: Artificial intelligence
CESM: Contrast-enhanced spectral mammography
CT: Computed tomography
EBM: Evidence-based medicine
EIBIR: European Institute for Biomedical Imaging Research
ESR: European Society of Radiology
EuroAIM: European Network for the Assessment of Imaging in Medicine
IPMN: Intraductal papillary mucinous neoplasm
MRI: Magnetic resonance imaging
RCT: Randomised controlled trial
SICUS: Small intestine contrast ultrasonography
US: Ultrasound
